# A novel PM motor with hybrid PM excitation and asymmetric rotor structure for high torque performance

**DOI:** 10.1063/1.4978400

**Published:** 2017-03-07

**Authors:** Gaohong Xu, Guohai Liu, Xinxin Du, Fangfang Bian

**Affiliations:** 1 School of Electrical and Information Engineering, Jiangsu University, Zhenjiang 212013, China; 2 Jiangsu Key Laboratory of Drive and Intelligent Control for Electric Vehicle, Zhenjiang 212013, China

## Abstract

This paper proposes a novel permanent magnet (PM) motor for high torque performance, in which hybrid PM material and asymmetric rotor design are applied. The hybrid PM material is adopted to reduce the consumption of rare-earth PM because ferrite PM is assisted to enhance the torque production. Meanwhile, the rotor structure is designed to be asymmetric by shifting the surface-insert PM (SPM), which is used to improve the torque performance, including average torque and torque ripple. Moreover, the reasons for improvement of the torque performance are explained by evaluation and analysis of the performances of the proposed motor. Compared with SPM motor and V-type motor, the merit of high utilization ratio of rare-earth PM is also confirmed, showing that the proposed motor can offer higher torque density and lower torque ripple simultaneously with less consumption of rare-earth PM.

## I. INTRODUCTION

The interior permanent magnet (IPM) motor is extensively applied in electric vehicles due to excellent merits, such as high torque power density, high efficiency, and wide speed range.^1^ With the increasing cost and unpredictable supplement of rare-earth PM, a series of rare-earth PM less or even free motor are researched as candidates, like switched reluctance motor (SRM), synchronous reluctance motor (SynRM) and PM-assisted synchronous reluctance motor (PMASynRM).^2–4^ However, in these motors, it is difficult to balance high torque density, low torque ripple and low consumption of rare-earth PM.^5,6^

This paper proposes a new PM motor with hybrid PM excitation and asymmetric rotor structure, offering high torque density and low torque ripple. In order to produce higher torque with lower consumption of rare-earth PM, hybrid PM material assisted with ferrite is utilized.^7^ Furthermore, the SPM is shifted to increase torque density and reduce torque ripple.^8^ Topology and features of proposed motor is presented in section II. In section III, the mechanical shifting angle of SPM is designed and optimized. Evaluation and explanation of improved performances of the proposed motor are offered in section IV. Finally, the conclusion is drawn in Section V.

## II. TOPOLOGY AND FEATURES

**FIG. 1.**
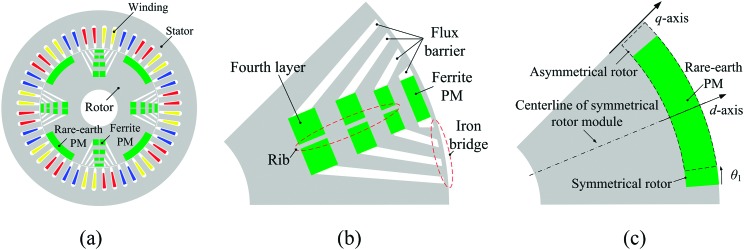
Topology. (a) Proposed motor. (b) PMASynRM rotor module. (c) SPM rotor module.

Fig. 1(a) shows the proposed novel PM motor. It can be seen that the rotor incorporates PMASynRM rotor module and SPM rotor module adjacently. The PMASynRM rotor module is assisted with ferrite PM and has four-layer flux barriers as shown in Fig. 1(b). Besides, the SPM rotor module is assisted with rare-earth PM. Then, the consumed rare-earth PM of proposed motor is reduced because of hybrid PM excitation. Furthermore, the PMASynRM rotor module can produce large reluctance torque and the SPM rotor module is employed to improve PM torque, resulting in smaller volume of rare-earth PM with the same output torque. Finally, as shown in Fig. 1(c), the rare-earth PM of SPM rotor module can be shifted to make asymmetric rotor structure for improving torque performance. The main parameters of proposed motor are summarized in Table I.

**TABLE I.** Main parameters of proposed motor.

Parameters	Value	Parameters	Value
Number of stator slots	48	Maximum current value (A)	236
Number of rotor poles	8	Number of turns per coil	11
Outer diameter of stator (mm)	264	Ferrite PM volume (cm^3^)	63.8
Inner diameter of stator (mm)	161.9	Rare-earth PM volume (cm^3^)	96
Outer diameter of rotor (mm)	160.4	Remanence of ferrite PM (T)	0.4
Inner diameter of rotor (mm)	50	Coercive force of ferrite PM (kA/m)	-188
Air-gap length (mm)	0.75	Remanence of rare-earth PM (T)	1.23
Stack length (mm)	50.8	Coercive force of rare-earth PM (kA/m)	-890

## III. DESIGN OF SPM SHIFTING ANGLE ON TORQUE PERFORMANCE

**FIG. 2.**
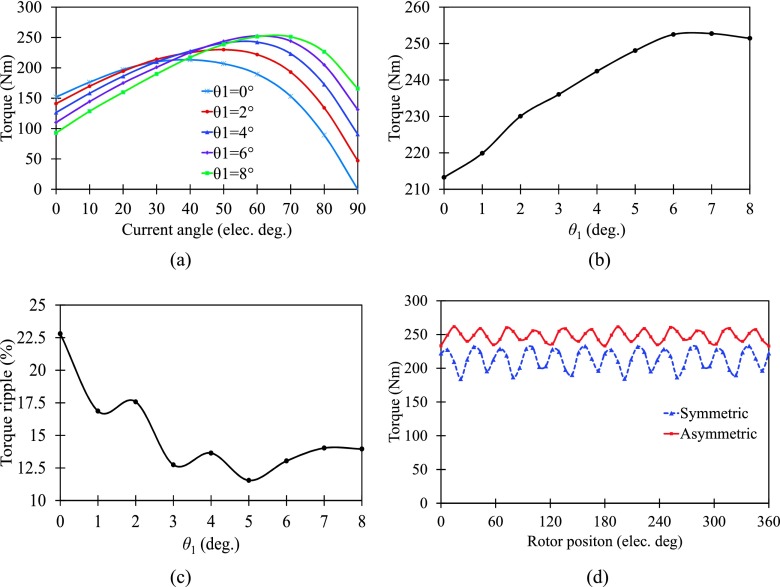
Torque performances. (a) Variation of average torque with current angle. (b) Maximum average torque with SPM sifting angle. (c) Torque ripple with SPM sifting angle. (d) Instantaneous torque.

In order to analyze how the torque performance is improved with the mechanical shifting angle of SPM, the time-stepping finite-element method (FEM) is employed. The shifting angle $\theta_{1}$ of SPM is marked in Fig. 1(c), which shows that the SPM shifting will cause the axis of the SPM no longer aligned with *d*-axis (axis of A-phase). When the SPM is shifted along the circumference of the rotor, the rotor is asymmetric and the variation of average torque with current advanced angle is investigated in Fig. 2(a). It is shown that the torque performance changes obviously when the SPM is shifted. There are three main characters can be achieved with the increase of $\theta_{1}$. First, average torque at the current advanced angle of 0° decreases. Second, torque at the current advanced angle of 90° increases dramatically. Third, torque under maximum-torque-per-ampere (MTPA) operation is improved significantly. In Fig. 2(b), the effect of SPM shifting angle on maximum average torque is investigated. When $\theta_{1}$ varies from 0° to 6°, the maximum average torque increases because saturation in *d*-axis is alleviated. Contrarily, the maximum average torque decreases when $\theta_{1}$ increases from 7° to 8° due to the reduced flux linkage of *d*-axis. Fig. 2(c) shows the variation of torque ripple at MTPA operation as function of $\theta_{1}$. It has to be pointed out that torque ripple varies nonlinearity considerably as $\theta_{1}$ increasing from 0° to 8°. Nevertheless, minimum torque ripple is obtained when $\theta_{1}$ equals 5°. Especially, the evident increase of torque ripple as the shift angles equals to 4° and 6° will be explained in section IV. As shown in Fig. 2(b) and Fig. 2(c), the SPM shifting is very effective in increasing the average torque and reducing torque ripple. Considering a compromise between average torque and torque ripple, $\theta_{1}$ is chosen to be 5° for the next evaluation. Fig. 2(d) shows the torque performance of the proposed motor with symmetric rotor ($\theta_{1}$ is 0°) and asymmetric rotor ($\theta_{1}$ is 5°). Compared with symmetric rotor motor, average torque of asymmetric rotor motor is significantly improved from 213.3 Nm to 248.1 Nm (an increase of 16.3%), and the torque ripple decreased dramatically from 22.8% to 11.5% (a reduction of 11.3%). It can be concluded that asymmetric rotor motor exhibits a significant higher torque performance in terms of both average torque and torque ripple over symmetric one without increasing consumption of rare-earth PM material.

## IV. EVALUATION AND EXPLANATION OF IMPROVED PERFORMANCE

**FIG. 3.**
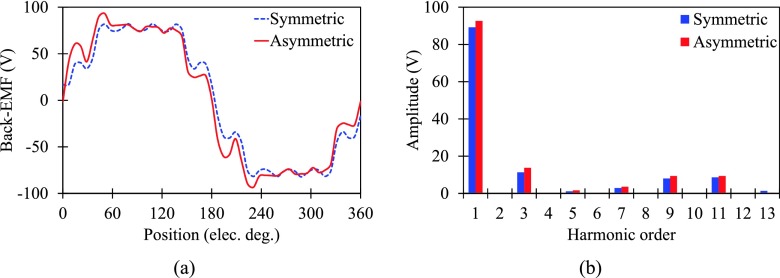
Comparison of no-load back-EMFs. (a) Waveforms. (b) Harmonic content.

Firstly, the no-load back-EMFs of the proposed motor with symmetric and asymmetric rotor at 1500 r/min are compared in Fig. 3(a). It can be observed that the back-EMF of asymmetric rotor motor has asymmetric crest due to the asymmetric rotor configuration. Moreover, Fig. 3(b) shows that the amplitude of fundamental component of asymmetric rotor motor is higher, verifying that the SPM shifting is benefit for higher PM flux linkage.

**FIG. 4.**
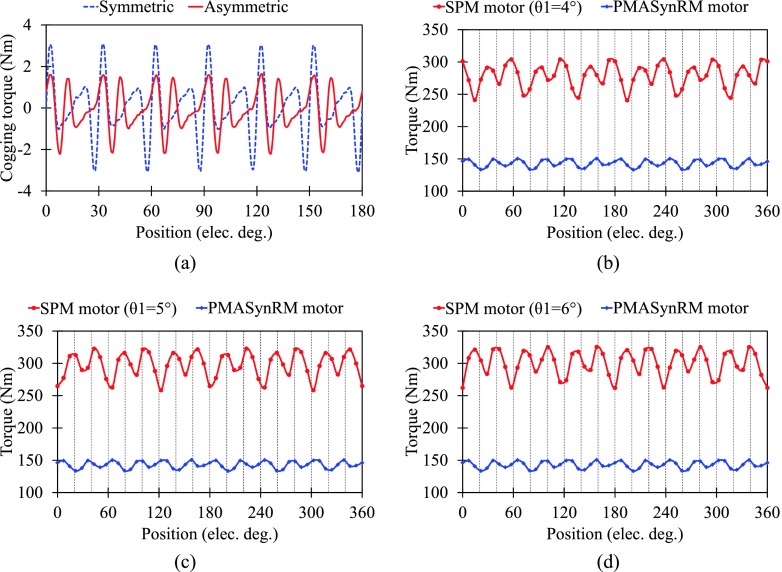
Torque ripple reduction. (a) Cogging torque. (b) Instantaneous torque with $\theta_{1}$=4°. (c) Instantaneous torque with $\theta_{1}$=5°. (d) Instantaneous torque with $\theta_{1}$=6°.

Secondly, the reasons for significant reduction of torque ripple can be analyzed from two aspects. The cogging torques of proposed motor with symmetric and asymmetric rotor are compared in Fig. 4(a). It can be observed that the peak to peak value of asymmetric rotor motor is only 62% of that of symmetric rotor motor. As shown in Fig. 4(b)–Fig. 4(d), it can be seen that there is phase difference of the instantaneous torque between the SPM motor (fully consists of SPM rotor modules) and the PMASynRM motor (fully consists of PMASynRM rotor modules). By shifting the SPM pole, the phase of torque of SPM motor shifts. When the $\theta_{1}$ equals 5°, the minimum torque ripple is obtained because of two reasons. First, the average torque under $\theta_{1}$=5° is larger than that under $\theta_{1}$=4°. Second, the position between the maximum torque of PMASynRM motor and the minimum torque of SPM motor under $\theta_{1}$=5° is closer than that under $\theta_{1}$=6° though their average torque is almost same. Hence, a significant reduction of torque ripple can be obtained when the two different rotor modules are combined together appropriately in the proposed motor.

Finally, the contribution sources for torque promotion are investigated. The output average torque, consisted of both PM torque and reluctance torque components, is improved significantly after optimization. Based on the definition of the *d*- and *q*-axes of this motor in Fig. 1(c), the flux linkage can be given as (1) and (2) in *d-q* coordinate system.^9^ Different from the traditional PM motor, the PM flux in *q*-axis should be considered due to the SPM shifting. Furthermore, the average torque can be calculated by (3). Then, the PM torque can be derived from (4). Finally, the reluctance torque can be computed by the difference between the average torque and the PM torque as given in (5).

$$\psi_{d}=\psi_{md}+\psi_{id}=\psi_{md}+L_{d}i_{d}$$ (1)

$$\psi_{q}=\psi_{mq}+\psi_{iq}=\psi_{mq}+L_{q}i_{q}$$ (2)

$$T_{e}=\frac{3P}{2}\left(\psi_{d}i_{q}-\psi_{q}i_{d}\right)$$ (3)

$$T_{pm}=\frac{3P}{2}\left(\psi_{md}i_{q}-\psi_{mq}i_{d}\right)$$ (4)

$$T_{r}=\frac{3P}{2}\left(L_{d}-L_{q}\right)i_{d}i_{q}=T_{e}-T_{pm}$$ (5)

where $\Psi_{d}$ and $\Psi_{q}$ are the total flux linkage of the *d*- and *q*-axes, $\Psi_{md}$ and $\Psi_{mq}$ are the PM flux linkage of the *d*- and *q*-axes, $\Psi_{id}$ and $\Psi_{iq}$ are the *d*- and *q*-axes flux linkage of current excitation, *i*_*d*_ and *i*_*q*_ are the currents of the *d*- and *q*-axes, *L*_*d*_ and *L*_*q*_ are the inductances of the *d*- and *q*-axes, respectively. *T*_*e*_ is the electromagnetic torque, *T*_*pm*_ is the PM torque, *T*_*r*_ is the reluctance torque, *P* is the number of pole pairs.

**FIG. 5.**
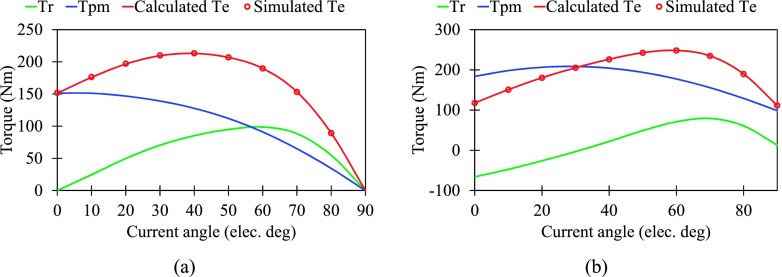
Torque characteristics versus current angle. (a) Symmetric rotor motor. (b) Asymmetric rotor motor.

**FIG. 6.**
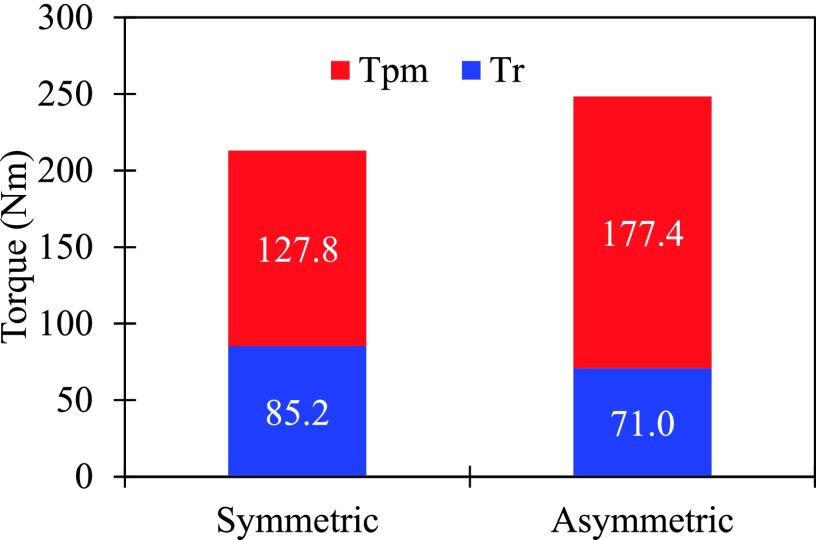
Torque components at MTPA operation.

The variation of torque components and total torque with current advanced angle is obtained and shown in Fig. 5. It can be seen that the simulated torque by FEM is almost the same as the calculated one by (3), with either symmetric rotor or asymmetric rotor, respectively. Besides, the SPM shifting causes the change of corresponding current angle of torque components and total torque. Furthermore, the corresponding torque components at MTPA operation are compared in Fig. 6. For the slight decrease of reluctance torque of asymmetric rotor motor, it is worthwhile to be noted that the promotion of output average torque is mainly contributed by the increase of PM torque, which has up to 39%.

**FIG. 7.**
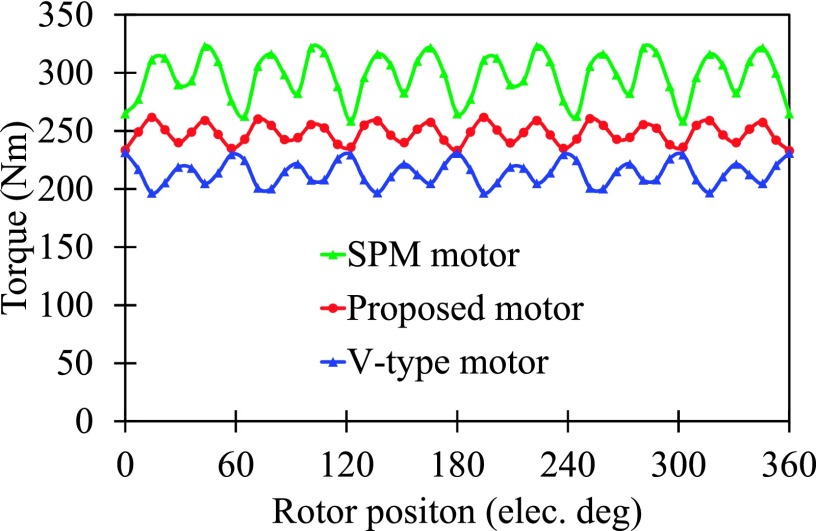
Torque comparison.

The instantaneous torques of proposed motor, SPM motor and V-type motor^1^ are given in Fig. 7, and the output torque and consumption of rare-earth PM volume are listed in Table II. It can be seen that the average torque of SPM motor is 297 Nm and that values in proposed motor and V-type motor are 248.1 Nm and 214 Nm, respectively. Although the torque density of SPM motor is the highest with the same dimensions in these motors, the consumed rare-earth PM volumes in SPM motor, V-type motor and proposed motor are 192 cm^3^, 104.6 cm^3^ and 96 cm^3^, respectively. Then the average torques per unit rare-earth PM volume in SPM motor, V-type motor and proposed motor are 1.55 Nm/cm^3^, 2.05 Nm/cm^3^ and 2.58 Nm/cm^3^, respectively. Hence, the proposed motor has the highest utilization ratio of rare-earth PM material in these motors, producing higher torque with less consumption of rare-earth PM material.

**TABLE II.** Quantitative comparison of torque in three motors.

Parameters	Proposed motor	V-type motor	SPM motor
Average torque (Nm)	248.1	214	297
Torque ripple (%)	11.5%	16.4%	21.7%
Rare-earth PM volume (cm^3^)	96	104.6	192
Torque/rare-earth PM volume (Nm/cm^3^)	2.58	2.05	1.55

## V. CONCLUSION

In this paper, a high torque performance PM motor with hybrid PM excitation and asymmetric rotor structure has been proposed and examined. By combining the PMASynRM rotor module and SPM rotor module, the SPM shifting angle of proposed motor can be designed to increase torque density and reduce torque ripple. Meanwhile the improved torque performance has been evaluated and analyzed by FEM. Furthermore, the reasons for torque promotion and ripple reduction have been analyzed and explained to confirm the improvement of proposed motor. Finally, the average torque per unit rare-earth PM volume of proposed motor has been verified as the highest, followed by V-type motor and SPM motor, successively.
